# On the Management of Labor Characterized by Defective Uterine Action, and the Comparative Value of Ergot of Rye and Galvanism in Obstetric Practice

**Published:** 1853

**Authors:** Robert Barnes

**Affiliations:** M. D., Lond., Lecturer on Midwifery to the Royal Free Hospital Medical College; Devonshire-square


*On the Management of Labor Characterized by defective Uterine Action, and the Comparative Value of Ergot of Rye and Galvanism in obstetric Practice.*—By Robert Barnes, M. D., Lond., Lecturer on Midwifery to the Royal Free Hospital Medical College.

In the range of obstetric practice there is a large class of cases which are characterized by defective uterine action. In many of these cases the object of the practitioner is to excite, by artificial means, that action which has ceased to be exerted under the spontaneous operations of nature. Two questions are thus at once brought before us, both of momentous importance. Firstly, what are the conditions of uterine inaction which call for interference? Secondly, what are the agents to be preferred for the purpose of evoking the dormant energy of the uterus?

The practical object of this paper forbids me to enter at any length upon the discussion of the first question, although the consideration of this ought logically to precede that of the second. An examination of this question befitting its importance would carry us through the almost entire history of parturition; not only this, but an inexhaustible field for controversy would be opened. It is not in every case in which labor is arrested on account of the diminution or cessation of uterine action that a recourse to means intended to rouse the uterus to exertion is necessary or proper. I can only refer to these leading circumstances which will best serve to illustrate the particular subject of this paper.

Beginning with the earliest occasion in the course of pregnancy in which the question of the means of exciting uterine contraction comes before the practitioner, I may refer to those cases in which it is considered desirable to anticipate the normal epoch of parturition. Although it cannot, with strict propriety, be said that cases of pregnancy complicated with distorted pelvis, or other conditions which render it hazardous to suffer gestation to be protracted to the full time, are cases marked by defective uterine action, still remembering that our principal object is to determine what agents are the best adapted to the purpose of exciting uterine action, we may usefully include those conditions which are held to indicate the propriety of inducing premature labor.

The condition next in order is defective uterine action at the commencement of labor, or in the first stage—a condition which may arise from a variety of causes. Then there is the state of uterine inaction in the second stage of labor, in which the evolution of the child is arrested for want of sufficient propelling power. Then we have those cases of defective energy in which the placenta is retained, and those in which hemorrhage occurs or is apprehended.

I have already said that defective uterine action does not always indicate a resort to the ordinary means of stimulating the uterus. There are no occasions in obstetric practice in which a nicer discrimination—a more accurate diagnosis—is required, before deciding upon the means of relief, than in those cases where the contractile energy is at fault. Our choice must frequently lie between the use of agents calculated to excite contraction, and those which have a directly opposite effect. We are frequently called upon to determine whether it be better to rouse the energies of the uterus, or to resort to manual or instrumental assistance. Upon our interpretation of the symptoms, and our appreciation of all the circumstances of the case, our selection of the mode of interference will depend; and upon this selection may hang the safety or the destruction of the patient.

If it be difficult to solve the preliminary question, whether we should endeavor to excite the uterus to action or not, how cautious ought we not to be in our choice of the particular means for inducing contraction, when that course is determined upon?

### I.

### *The action of Ergot of Rye, and the objections to its use.*

There is one agent frequently—much too frequently—resorted to on account of its power of exciting uterine contraction, the ergot of rye. A principal object of this paper will be to exhibit the dangerous properties of this drug; to show what little mastery we have over its action when once administered, and the consequently fatal results attending an error in diagnosis—a mistake in the application of the drug. If I further succeed, as I hope to do, in proving that we possess another agent at once more effective, more manageable, and more safe, and capable of useful application in all those cases in which ergot of rye is commonly employed, I shall not have uselessly engaged the time of the reader.

The ergot of rye is capable, under certain circumstances, of producing the most marked and decisive effect in exciting the uterus to contract. An agent possessing such a power, it need not be said, is liable to abuse. It is notorious that many practitioners carry this drug in their pockets, esteeming it an indispensable adjunct to the practice of midwifery. It is amongst midwives—necessarily the most ignorant of obstetric practitioners—that this custom chiefly prevails. That this should be so is most deeply to be deplored. No agent, no species of interference in natural parturition, supplies more frequent or more distressing illustrations of that maxim, the most trite, the truest, and the most neglected, "A meddlesome midwifery is a bad midwifery," than does the ergot of rye. For one woman who has derived substantial benefit from its use at the time of labor, it may be confidently assumed that one hundred have found reason in long-enduring subsequent sufferings to rue the hour when they were made to swallow the nauseous draught under the delusive promise of a speedy release from pangs, hard indeed to bear, but mostly beneficial in their result.

In discussing the uses of ergot in obstetric practice, I think it more convenient to postpone the consideration of its use in inducing premature labor, and to refer in the first place to its employment in labor at the full time. When ergot is administered before the expulson of the child, the effects are usually as follows:—in virtue of its peculiar property of exciting contraction of the uterus, in about fifteen or twenty minutes the uterus is perceived to be under the influence of the drug. A spasmodic contraction begins in the uterine muscular fibres. Whether this is excited by the direct stimulus of an ergotic element carried in the blood to the uterus, and thus acting immediately upon the uterine nerves or muscular fibres, or whether the ergotic element acts primarily upon the spinal marrow—that is, whether the first step in ergotic labor is of eccentric or of centric origin—it is not easy to determine. But it is quite certain that when once the contractile energy of the uterus is roused, other actions, violent in proportion to the efforts of the uterus, are brought into operation. Secondary disastaltic or reflex action of the expiratory muscles is induced with a violence in direct relation to the violence of the primary uterine contraction. If there be no invincible obstruction to the expansion of the mouth of the womb and the expulsion of the child, the child will be driven with precipitate fury through the pelvis and os externum, at the imminent risk, however, of lacerating the perinæum, which has had no opportunity of expanding gradually and safely, as it does before the normal pressure of a labor completed by the natural powers. It should be respected as a fundamental axiom in obstetrics, that as child-bearing is a natural function, so is its safe fulfilment insured by adequate contrivance. Each step in the long process of parturition—from the first action of the uterine muscular fibres which determines the expansion of the os uteri, to the final contractions which expel the placenta and close the open mouths of the uterine vessels—is only one of a gradation disposed according to a pre-ordained order, with a view to the final result. To invert or to disturb this order, as the use of ergot in natural labor can hardly fail to do, by anticipating the due period of the expulsive pains, is to disconcert all the arrangements of nature; to throw the whole process of parturition into confusion, to resign to the uncontrollable fury of spasmodic action that process which depends for its safe completion to mother and child on a regular co-ordination of physiological actions, in which each stage is essential to the proper progression of the succeeding one.

But if an unforeseen obstruction exist, then more terrible results must be apprehended. When an obstruction occurs in the course of labor, it frequently happens that nature takes the alarm; the uterus, so to express it, seems gifted with a kind of prescience that the obstacle is beyond its power to overcome. It therefore intermits or ceases those contractile efforts, which, if continued, would entail either rupture of its own structure, or impaction of the child: the contractions become abortive. Now, when ergot is given, it is presumed that a reason is discovered for its use in the intermission or cessation of the pains. This intermission or cessation may arise—

1st, From this prescient reluctance of nature to act in the face of a mechanical obstacle.

2nd, From exhaustion in consequence of long-continued unavailing efforts.

3d, Because the proper time for expulsive efforts has not yet come, and the proper physiological stimuli to disastaltic action have not come into operation.

Now, in every one of these cases the action of the ergot of rye is likely to be prejudicial: first, if in the case of an obstacle to the progress of the child, ergot be given, and its peculiar action ensue (which, fortunately, is not always), then the uterus contracting, and vainly contracting, upon the opposing force, is goaded by opposition into more furious efforts. Before its ungovernable struggles something must give way. Rupture of the womb is one probable termination; or the child may be jammed in an unfavorable position into the pelvis and there impacted, and convulsions and death may close the scene. All this I have actually witnessed.

When contraction has ceased from exhaustion, to what purpose will you lash the jaded uterus to renewed exertions of which it is incapable? In such a case ergot can manifestly have none but the most injurious effect. And yet it is in such cases that it is frequently resorted to.

It may be urged thst these are not fit cases for the use of ergot, that its injurious action here cannot be advanced as an argument against its employment in proper cases. This may be so. But then how difficult is the diagnosis— how fatal a mistake! And if ergot be a drug in such common and extensive use as it is known to be, and that amongst the most ignorant, how can we expect the diagnosis to be just, or that fatal errors shall not be frequently committed?

The uncontrollable action of the drug when once administered, added to the difficulty of diagnosis, constitutes the gravest objection against it. A mistake is irretrevable; once given, the case is as it were out of our hands. We know of no certain means of mitigating or counteracting its effects when they turn out to be violent or altogether injurious. I propose to pass in rapid review some of the proofs of the dangers attending the use of ergot in obstetric practice, dangers too much overlooked, if not ignored, by many.

I will first consider the dangers to the mother.

Rupture of the Uterus. Dr. Trask, who analyzed the histories of all the cases he found recorded, found that in a large proportion ergot had been given. It is quite true that in many of these cases the ergot was given in contravention of the rules usually laid down. In some there was obstruction to the labor from distortion of the pelvis, malposition, or malproportion. But this consideration does not diminish the value of the general fact, that ergot has frequently caused rupture of the uterus. If given before the head has descended into the pelvis, who can determine, even in the case of a well-formed pelvis, that an obstacle will not arise in the unusual or morbid enlargement of the head? If given even when the head is pressing on the perinæum, the os perfectly open, and all those conditions apparently present which are held to justify the resort to ergot, who can tell whether a second or third child may not be behind? And who would knowingly goad the uterus into spasmodic fury in a case of twins? How great is the probability that the second child would be driven into the pelvis in a transverse position? Even up to the moment when the head is about to emerge from the outlet the use of ergot then is not safe, and I shall presently show that it is not necessary.

The next accident is rupture of the perinæum. The danger of this accident is so obvious that I need not do more than record it.

Lacerations of the os uteri, sebsequent inflammation, and hypertrophy of the cervix, are events which I have frequently traced back to ergotic labor.

Prolapsus and procidentia of the uterus and bladder. These distressing affections are not unfrequently the secondary result of inflammation and hypertrophy of the cervix uteri; but even when not thus the indirect consequence of ergotic labor, they may result directly from the violent dislocation occasioned by ergotic contractions. I have known a striking case of this kind.

Case 1.—A woman had ergot given to her in a perfectly natural labor, to expedite delivery. It brought on one continued pain of a character and intensity such as she had never experienced before, and during which, to use her own expression, she felt as if "the whole of her body was coming from her." The child was violently extruded, and the uterus and bladder were driven down by the secondary excited action of the expiratory muscles into the pelvis, the bladder remaining outside the labia pudendi. It was not until some time had elapsed, and careful general and local treatment, that these organs were restored to their normal position.

Ergot may induce certain injurious effects upon the mother's system. Dr. Hardy* relates, that in "several cases where the circulation of the patient had undergone depression from the action of ergot, the effect continued for several days, notwithstanding in some instances inflammation of the uterus followed delivery, and the uterine tumor not unfrequently remained much larger than natural, even when there was no inflammation." Dr. Hardy also quotes the eminent authority of Dr. Johnson to the fact, that "that the volume of the uterus is often found much greater than after ordinary labors, imparting to the hand almost the feel of a uterus before the expulsion of the placenta."

Drs. Hardy and M'Clintock have observed a marked diminution in the frequency of the mother's pulse in from fifteen to twenty minutes after the administration of ergot. And all concur in noticing the dangerous depression following the use of ergot when given in cases where the powers of the system have been reduced by hemorrhage. In one such case ergot was almost immediately followed by most alarming symptoms, and depression requiring the most powerful stimulants. In several cases the depressed state of the circulation continued several days.

Dr. Ingleby relates the following case:†—"A highly-esteemed friend once found it necessary to pass his hand into the uterus to remove an adherent placenta, the ergot of rye having been previously administered. The introduction was carefully performed. The straining and opposition to his efforts on the part of the woman were exceed-

---

* Dublin Quarterly Journal, 1845.

† On Uterine Hemorrhage, p. 186.

ingly great, and at the moment when the operator's hand had reached the organ, my own hand making counter-pressure on the abdomen, the patient became violently convulsed, and died in less than a minute." The cause of the convulsion, Dr. Ingleby expressly states, was not loss of blood.

We will now consider the injurious effects of ergot upon the child.

Drs. Hardy and M'Clintock observed that the pulsations of the fœtal heart underwent a similar diminution in frequency to that witnessed in the mother, and that this was succeeded by irregularity and intermission, and that it became inaudible. Dr. Hardy, Dr. Beatty, and others, after careful observation directed to this point, assert, that unless the child be born within a limited interval from the administration of the drug, it will be still-born. The excessive mortality of the children in ergotic labor is a fact well established, although disputed by some practitioners enthusiastic in the praises of ergot. The Prefect of the Seine had observed an almost regular annual increase in the number of still-born children, and he was informed that in a large number of these cases ergot of rye had been given during labor. He put the following question to the Academy of Medicine:—"What may be the influence of ergot of rye on the lives of infants, and on the maternal life?" The report made by a commission of the Academy, consisting of Orfila, Adelon, Villeneuve, Merat, and Danyau, contained the following conclusion: "Ergot of rye administered improperly causes death to the fœtus, and injury to the mother." The immediate source of danger to the fœtus is either the toxical property imparted to the blood, or the interruption to the circulation through the uterus and the placenta, occasioned by the long-continued contraction of the uterus. In this latter case the child may perish from asphyxia. These are the usual sources of danger; but there is a third. The long-continued and violent pressure to which the child is subjected during ergotic labor may compress the brain beyond the limit of endurance, or it may impede the circulation through the umbilical cord. The toxical agency of the ergot upon the fœtal heart is exemplified in the observations already referred to of Dr Hardy. The influence of contraction of the womb in arresting the circulation through the placenta, and consequently the fœtal circulation, has been demonstrated to me by actual observation. The case is so interesting, and the opportunity of making a similar physiological experiment must be so rare, that I will cite it in detail.

Case 2.—A woman, with an extremely contracted pelvis, and who ten years before had been delivered by craniotomy by Dr. Waller, consulted me about her condition. She was again pregnant. I became satisfied of the propriety of inducing premature labor; and the agent I determined upon employing was galvanism. Having waited until it was estimated that seven months of gestation had passed, the operation was commenced. I shall have to relate presently the course of the labor under the use of galvanism, and may therefore pass at once to the particular point it is my present wish to illustrate. When labor had set in, and the os uteri was partially expanded, the cord came down into the vagina. The pains being of a languid, uncertain character, the galvanic stimulus was kept up. The pulsations of the cord were strong, and 80 in the minute. Galvanism was applied during the pains; the contractions were sensibly increased in force, and during the contractions the pulsations in the cord became intermitting, and occasionally stopped. As the pain went off, and as the galvanism was discontinued, the pulsations resumed their former strength and regularity. I then tried the effect of galvanism in the absence of a pain. Contractions were induced, and the intermittence of the pulse followed.

I then observed the effect of a pain uninfluenced by galvanism. The intermittence of the pulse was the same. I repeated these observations several times, and always with the same result. Towards the termination of the labor, a strong expulsive pain came on, during which the head, which was very small, was driven into the vagina, without, however, causing any pressure upon the cord. During the strong pain the pulsation in the cord stopped entirely, but returned when the pain went off.

But fœtal circulation is arrested during the physiological contraction of the womb for a short time only, and is completely restored during the intervals sufficiently long to insure the safety of the child. In ergotic contraction the interruption is total, unremitting, and protracted. Shall we wonder if the child occasionally perishes from asphyxia?

Dr. Ramsbotham, whose experience in the use of ergot in inducing premature labor is probably greater than that of any other practitioner, says: "After a great number of trials I observed that altho' the mothers recovered as well as if through an ordinary labor, their systems not being in any sensible degree injuriously affected by the drug, yet that the proportion of children born still was greater than when the membranes were punctured. This I attributed to the baneful influence of the medicine upon the fœtus." Dr. Ramsbotham modified his practice in consequence. He further says that "Wright's experiments prove decisively that medicine has a most prejudicial influence upon the young *in utero*, even to their destruction?"

If the child survives the perils of ergotic labor, is it free from subsequent danger.

Dr. Ramsbotham says: "It has happened to me in four different instance to witness the death of the fœtus, a few hours after birth, by convulsions, after the induction of premature labor by ergot."

These facts, which might be greatly multiplied, prove beyond a doubt that ergot of rye is capable of exerting the most deplorable and even fatal mischief both upon the mother and the child. It follows from this circumstance, the uncontrollable nature of its action, and the difficulty that exists in many instances of forming an accurate diagnosis, that the use of the ergot of rye in obstetric practice should be reduced within the narrowest possible limits. I believe that the restrictions to its use must be carried very much further than is generally prescribed. It is a matter of extreme doubt to me whether it should ever be administered before the child is born; and in cases of uterine inertia after delivery, accompanied with retention of the placenta or hemorrhage, it has been shown to be by no means free from objection. But the most effectual way of attaining the object I propose, of minimizing the use of ergot in obstetric practice, is to show that we possess other means at once more safe, more effectual, and capabls of successful application in all those cases in which the ergot has been recommended.

### II.

### *The Uses of Galvanism in Obstetric Practice.*

I pass over all those means of rousing the uterus from a state of inertia which occur to the mind of the practitioner conversant with the physiology of parturition. An encouraging word, the restoration of hope and confidence, a timely stimulating draught, the pressure of the hand upon the uterus externally, the use of cold variously applied; all these, and a thousand other means may, in numberless cases, supersede the resort to ergot. It is my object to direct attention to an agent which is, I believe, an absolute substitute for ergot, and one which may be resorted to with confidence in every form of labor marked by defective uterine action, and in every case where it is desired to excite contraction of the uterus. The agent which answers to these conditions is galvanism.

From time to time many valuable but isolated observations upon the use of galvanism in different cases of obstetric practice have been published. But no systematic attempt has been made to prove that in galvanism we possess an agent capable of universal application wherever we require a safe and effectual stimulus to the muscular structure of the uterus. I shall consider the uses of galvanism in the successive epochs of gestation and parturition, beginning with its use in the induction of premature labor.

### III.

*The Use of Galvanism in the Induction of Premature Labor.*

In 1803, Herder* suggested the use of electro-galvanism for the induction of premature labor. In August, 1844, Drs. Horninger and Jacobi† succeeded in bringing on labor by the electro-galvanic apparatus after other means had failed. The application was immediately followed by uterine action, and the child was born in an hour from the commencement of the operation. A successful case under the hands of Mr. Demsey is also referred to by Dr. Golding Bird. My researches into what has been written on the subject have not been sufficiently minute to enable me to say that no other similur cases have been recorded. In January, 1851, I myself had no opportunity of testing the efficacy of this agent.

Case 3.—I have already referred to this case for the purpose of illustrating the effect of contraction of the uterus upon the fœtal circulation. The result, although perfectly satisfactory, was by no means so speedily accomplished as in the case of Horninger and Jacobi. I had previously endeavored to bring on labor by puncturing the membranes, and inserting a sponge-plug in the cervix uteri. This proceeding was followed by no symptom of labor. On the 23d of January I applied the galvanic battery for half an hour, placing one pole on either side of the uterus. Immediately after commencing the shocks the bladder was

* Diagnostische Practische Beitrage zur Erweiterung der Geburtshulfe, Leipsig. 1803.

† Busch's Neuve Zeitschrift fur Geburtskunde, vol. xvi.

irresistibly emptied, to the evident annoyance of the patient. The womb was felt to become hard, and the patient herself was sensible of contractions and increased movements of the fœtus. The contractions did not continue on the cessation of the galvanism, and I therefore repeated the applications on the 24th and 26th, for about an hour each time. On the 26th a "show" took place. On the evening of the 27th, slight pains were felt; the cord was presenting, a small loop coming through the os uteri, which was now dilated to the size of a shilling, but feeling rigid. She had had rather copious flooding in the day-time, but it had stopped. The head was felt lying on the pubes in front of the os uteri, the cord coming down in the free space behind it. On the morning of the 28th, the galvanism having been applied at intervals all night, the pains had increased. I have already mentioned how the galvanism increased or originated contraction. At nine A. M. the child was born. It was apparently not more than six moths old. The patient had certainly reckoned falsely. The child's heart was pulsating, the chest made three or four convulsive heaves, at which the mouth opened, but no air seemed to enter: the lungs refused to expand; the walls of the chest were drawn in towards the spine. I endeavored to excite respiration by the galvanic apparatus, but although I could at will cause a respiratory effort, the child was evidently too immature to live. The womb contracted favorably, and the placenta being withdrawn was found healthy. The patient recovered without a bad symptom.

The excellent effect of galvanism in this case led me to recommend the use of the same agent to my friend, Mr. Mansford, who has favored me with the following account:—

Case 4.—"The lady, whose case led me to attempt the induction of premature labor, was in the forty-first year of her age, and the thirtieth week of her fifth pregnancy. On the 8th of November, 1852, having ruptured the membranes, I introduced one wire of the apparatus within the os uteri, and placed the other in contact with the spine. From the one introduced in the uterus I had removed the brass handle, and twisted the wire upon itself so as to form a loop sufficiently curved to insure its remaining steadily in its proper place. I also carefully enveloped a considerable portion of this wire with lint, as well to protect the vagina from the twisted portion and extremity, as to prevent the galvanic current from being diverted from the uterus. I then increased its power until it produced 'the most severe cutting pains in the loins,' 'great bearing-down,' and 'a dreadful commotion in the womb.' These were my patient's own expressions. This operation was repeated on the 9th and 10th, each morning for half an hour; the effect, however, had not been as yet altogether satisfactory, as I had not been able to maintain a continuous action, but on the fourth morning—viz, the 11th—I remedied this defect, and kept up a continuous current for three-quarters of an hour, when my patient begged me to desist, which I did, and determined to wait a few days to see if this might accomplish the desired effect. Happily on the 14th, without any further interference, labor commenced, and terminated within four hours, in the birth of a living child, and not a single untoward symptom occurred spontaneously. It was altogether a most satisfactory case."

The foregoing results are directly at variance with the opinion of Dr. Golding Bird, who says—"The result I have arrived at is, that this agent, like the ergot of rye, and perhaps other ecbolic remedies, generally fails to develop uterine action *de novo*. . . . Hence, though I believe it will generally fail to induce premature labor, it will as generally succeed in stimulating the uterus to vigorous contraction after labor has actually commenced." In weighing this negative opinion, it should, however, be observed, that the latitude of qualification implied in the word "*generally*," deprives it of all precision of meaning.

It would lead me beyond my present purpose to discuss the relative advantages of galvanism, and the douche recommended by Dr. Kiwisch, and other methods. I will simply remark, that whatever method be determined upon for the purpose of bringing on labor, the stimulating property of galvanism upon the uterus will be a most useful adjuvant.

I will brifly refer to the great superiority of this method over the use of ergot of rye. An unexpected obstacle to the expulsion of the fœtus may arise after the administration of ergot; there is, consequently, danger of rupture of the uterus. How, for example, can we foretell that the child will not be driven into the pelvis in a transverse position? Secondly, there is the great improbability that the child will be born within any reasonable period after the administration of ergot; many doses are required; there is the risk of ergotism to the mother; and the peril to the child rises in proportion to the amount of ergot given; moreover, it is extremely uncertain whether the ergot will act at all.

### IV.

*The Use of Galvanism in Inertia during the First and Second Stages of Labor.*

I will now illustrate the effect of galvanism in lingering labor from uterine inertia. An interesting case of this nature is recorded by Mr. Cleveland,* which was brought to a close within fifteen minutes after the use of the electro-galvanic apparatus had commenced: Mr. Houghton† also relates four cases of arrested labor from atony of the uterus brought to a successful termination by the agency of galvanism. In three of these ergot had previously failed.

In a similar case I have myself experienced the like good effect, but I prefer citing the following account supplied to me by my friend Dr. Mackenzie:—

---

* Medical Gazette, June 1845.

† Dublin Quarterly Journal, February 1852.

Case 5.—"I was sent for one morning to a young woman who had been admitted in labor at the Paddington Infirmary, and on examination I found that the head presented. Although she had been several hours in labor, the os uteri was but little dilated. I saw her in the course of the same afternoon, but still found very little dilatation.

"At ten P. M., but little progress had been made. I now determined to try the effect of galvanism, and applied one pole of a single current machine to the spine, and the other, by means of Radford's director, to the neck of the uterus. The current was from time to time intermitted, and uterine action of a vigorous character was excited. In about an hour, a fine, living child was born. So vigorous were the expulsive efforts during the passage of the head through the os externum, that I was obliged to take particular pains to prevent rupture of the perinæum. The impression left on my mind by this case was, that galvanism should not be employed except very cautiously in primiparœ, or in any other instance in which the perinæum is rigid or imperfectly developed."

Galvanism may also be usefully employed in many cases of hemorrhage before the birth of the child.

A judicious application of this agent may, in many cases of arrest of the head from inertia, obviate the necessity of resorting to the use of the forceps.

### V.

### *The Use of Galvanism in the Third Stage of Labor and Hemorrhage.*

We posses a greater amount of evidence of the value of galvanism in the third stage of labor. Dr. Radford has contributed many valuable observations, exemplifying the power of galvanism in exciting contraction of the uterus in cases of post-partum hemorrhage. These are too well known to require to be cited. Mr. Houghton has added other cases which occurred under his own observation.

The only instance I will adduce here is one which occurred recently to Dr. Mackenzie.

Case 6.—"The patient had been upwards of forty-eight hours in labor, under the care of Dr. Keogh, who had called in Mr. Clark, by whom I was sent for. When I saw the patient, uterine action had entirely ceased, and I found, on examination, that the head was impacted in the pelvis, the face presenting with the chin to the left cotyloid cavity. As the patient was exhausted, an opiate had been given, and as she was disposed to sleep, we agreed to meet again in some hours, and if uterine action did not return, to deliver by the forceps. At the appointed time no return of uterine action had taken place. I applied the forceps; the operation was accomplished with extreme difficulty, and the woman was delivered of a fine, large, living child. I left the patient shortly asterwards, but the next day, on meeting Dr. Keogh and Mr. Clark, I learned that great apprehension had been felt throughout the night as to the occurrence of hemorrhage, inasmuch as the uterus had remained flaccid and uncontracted, and at the time of my visit it reached above the umbilicus, and was very soft and flabby. I proposed galvanism, and applied one pole to the spine and the other to the neck of the uterus, occasionally intermitting the current. This was done for half an hour, and evident uterine action was excited, the uterus becoming harder and smaller, and on removing the poles two large coagula were expelled. The next day the uterus was more contracted and smaller, and no hemorrhage had occurred. Galvanism was again used for half an hour. The uterus certainly contracted under its influence. The following day no hemorrhage had occurred, and the condition of the uterus was such as not to require any further recourse to the agent. The woman from this time recovered in a most favorable manner." Dr. Mackenzie adds the following remarks, in which I entirely concur: "It appears to me that the results of galvanism in this case were highly satisfactory, because coagula retained in the uterus, from atony of the organ, are not only calculated to occasion hemorrhage, but by undergoing a species of putrefactive decay, to give rise to fever and all the consequences of vitiation of the blood. Under such circumstances, I have known the hand forcibly introduced into the uterus many days after labor for the removal of such coagula, with very disastrous results—results which this case shows may be obviated by having recourse to galvanism."*

### VI.

### *Other Uses of Galvanism in Obstetric Practice.*

There is another case of not unfrequent occurrence in obstetric practice; in which galvanism may be of eminent service—temporary paralysis of the bladder following delivery. A case I have already related illustrates the power of galvanism in causing contraction of the bladder. Drs. Goodwin and Radford† describe an interesting case, in which the catheter was employed two or three times a-day, and could not be dispensed with. On Dr. Goodwin's suggestion galvanism was tried, and the first application proved successful.

I would especially recommend the use of galvanism in those cases in which the action of the uterus has been unfortunately paralyzed under the influence of chloroform. In such cases I believe no other stimulus that can be applied will answer with equal certainty or efficiency.

I am also sanguine as to the value of galvanism in exciting respiration in asphyxiated children.

There is another class of cases in which galvanism promises to be of the greatest service. A most interesting case has been recorded in which Dr. Tyler Smith‡ was enabled

---

* Further observations are required in order to determine the action of galvanism upon the fœtus *in utero*. Present experience, however, does not indicate that it exerts any injurious effect.

† Provincial Medical Journal, December, 1844.

‡ The Lancet, 1852.

to produce expansion of the neck of the uterus, and to bring an intra-uterine polypus into view, so as to admit of the application of a ligature, by the application of galvanism after ergot had failed. I have also employed it with success for the purpose of causing the expulsion of hydatids. This case occurred in connexion with my colleague, Mr. Forbes, and I will relate so much of the account as bears upon the question before us.

Case 7.—Ann W——, aged forty-two, had had eight children and three abortions. She applied to Mr. Forbes, on the 17th of June last, having anasarca of the legs. Two months before, she suffered a burning pain in the region of the womb. She had menstruated up to Christmas last. Since that date there had been a little hemorrhage discharge at intervals. For the last month there had been a continual discharge of colored fluid. Her health is much impaired, and her strength lowered. On the 18th, while in bed, she felt a vaginal discharge, and on getting up passed a large quantity of blood. The pulse was weak, thready, 108; face blanched; headache intense. No pain preceded the hemorrhage. There was a tumor in the seat of the pregnant womb, extending more to the right side, and reaching to the umbilicus; it was firm and elastic, tender on pressure, which did not bring on labor pains. The os uteri was the size of a shilling, and rigid. No placental murmur or sounds of fœtal heart heard. The breasts were quite flaccid. Os slightly expanded towards the afternoon. A dead fœtus, or some diseased condition of the ovum, was suspected. In consultation, Dr. Barnes suggested galvanism to cause contraction; this had the desired effect, and Mr. Forbes was enabled to bring down a bunch of hydatids. The vagina was then plugged, and the abdomen bandaged. The disposition to contraction thus given, more hydatids were afterwards passed. Tincture of ergot of rye was then given in small doses. Early on the morning of the 19th, the patient passed a large mass of hydatids, which was expelled suddenly, with a pain like that of labor. She was quite exhausted with loss of blood and previous disease; symptoms of inflammation appeared, and she sank the same night. The post-mortem examination revealed a large fibrous tumor in the walls of the uterus, and an advanced stage of granular degeneration of the kidney.

In such a condition of the uterus and the patient, none of the ordinary means of exciting contraction could have been employed with equal safety and advantage. The necessity of inducing contraction to expel the contents of the womb and arrest the hemorrhage was obvious, and the utility of galvanism in accomplishing this was manifest. I am disposed to regret that the galvanism was not more freely used. The expulsion of the hydatid placenta might have been hastened.

It is beyond the strict scope of this paper, but I may be permitted to refer to the advantages attending the use of galvanism in amenorrhœa, hysteria, and other diseases of females, advantages which have been clearly established by Dr. Golding Bird, Dr. Gull, and others. The stimulating influence of galvanism is well worthy of trial for the purpose of exciting the lacteal secretion.

### VII.

### *Mode of applying Galvanism.*

I have now gone through a series of illustrations affording evidence of the use and value of galvanism in most of the forms of labor characterized by defective uterine action, and in other cases where the indication is to excite the contractile property of the uterus. I will conclude this paper with a brief description of the mode in which this powerful agent should be applied, and a summary of the advantages it especially possesses in obstetric practice over the ergot of rye. The ordinary electro-magnetic apparatus in use for medical purposes is, I believe, the best form that can be employed. The principle of this apparatus consists in the induction of magnetic currents by a current of elcctricity, and the production of a rapid succession of feeble shocks by continual interruptions to the current. I have observed that the uterine contractions are always provoked at the break and renewal of the circuit. Repeated shocks act as a far more effectual and certain stimulus to uterine contractility than a continued current. It is probably through inattention to this fact that some practitioners have failed in effecting contraction of the uterus by means of galvanism. As to the mode of applying the poles, I do not think it necessary to apply one over the spine and the other to the neck of the uterus, as is usually done. I have found the application of the discs, covered with thin flannel moistened in water, one on either side of the obdomen over the uterus, much more convenient, and quite as effectual. The practice of applying one pole over the spine and the other to the neck of the uterus, further seems to me to be based upon an erroneous view of the mode in which galvanism acts upon muscular fibre. When the poles are thus applied, one to the spine and the other to the cervix uteri, it is doubtful whether the ensuing contraction of the uterus is due to primary excitation of the spinal marrow. It is proved by the experiments of Matteucci, and it is confirmed by general observation, that galvanism acts directly upon the muscular fibre, stimulating it to contraction. It is clear that this direct action can be as effectually obtained by passing the shocks through the uterus, as by placing the poles on either side of the abdomen. I would not be understood to affirm that this immediate action of galvanism upon the muscular fibre is its sole mode of action, but that it is the primary and essential one: this primary peristaltic action commenced, the secondary and tertiary diastaltic, emotional, and voluntary reactions upon the uterus follow.* The duration of

* It will be seen, from what has been said above, that I cannot concur implicitly in the views or conclusions of Dr. Golding Bird expressed in the following quotation:—"In the magneto-electric the application must depend upon the requirements of the case. It is often found that nothing but a primary excitation is wanted, and this being supplied, the uterus will go on contracting spontaneously. In those cases where it is required to originate uterine contraction, as in the induction of premature labor, several applications of an

---

coil in which currents are excited by repeatedly breaking contact by a vibrating bar, we have two currents moving in opposite directions, to each of which the patient is submitted. Now these curents are of unequal strength; and if the most energetic—that on breaking contact—be passed in the direction of the *vis nervosa*, it will produce painful contractions, which, the moment it passes in the opposite direction, will become relaxed; for a direct current tends to produce contraction; an inverse current, paralysis. Hence, I should urge the accoucheur not to employ the apparatus in which both these currents are produced, but simply the single current machine. In using this, I would suggest the positive conductor to be placed over the lumbo-sacral region, and the other to be carried by gentle friction over the abdominal surface. In this way, powerful uterine contractions may be easily produced."

Are we, when we place the positive conductor over the spine, and the negative one over the abdominal surface, warranted in assuming that we are passing a current in the direction of the *vis nervosa?* Are we so much as warranted in assuming that we are acting upon the contractility of the uterus through thn medium of nerves at all? Conclusions such as these should be based upon demonstration. Has the uterus been isolated from all surrounding textures, preserving only its connexions with the spinal and sympathetic nervous system; and have the effects of passing a "single current" along the nerves in a centripetal and centrifugal direction been observed? No. But this has been observed;—that muscular fibre, both voluntary and involuntary; will contract under the galvanic stimulus when its relations with the nervous centres are severed altogether! The only legitimate conclusion from the known facts appears to be that, whether the shocks of the single or double current machines be passed through the uterus in one direction or in the other, whether through the lumbo-sacral region and the abdomen or cervix uteri, or from side to side of the abdominal walls, the uterine muscular fibre is stimulated and will contract. The observations of Matteucci, upon which the reasonings of Dr. Golding Bird appear to be built, apply more especially to the effect of very feeble currents upon the motor and sensible nerves of the spinal system and the voluntary muscles.

hour's duration will be necessary. The uterus cannot be roused to perfect action before the appointed time without repeated stimulation.

### VIII.

*The Special Advantages of Galvanism as an Agent for producing Uterine Contraction.*

Among the advantages of galvanism more especially worthy of attention are—

1st. The simplicity of the operation.

2nd. The extensive range of cases in which it may be successfully employed, rendering the electro-magnetic apparatus a desiderable addition to the armamentarium of the obstetric practitioner.

3d. The perfectly manageable character of the agent. Its action may be broken off and renewed at pleasure. The moment we think the uterus is acting too powerfully under its use, we may instantly withdraw the exciting agency, and leave the uterus to the ordinary physiological stimuli, which seldom impel the organ to undue activity. It moreover admits of easy regulation; both the strength and duration of this agent are completely under our command. We have it in our power to imitate in a remarkable manner the natural pains both as to intensity and intermission. Ergot has neither measure nor certainty.

4th. Its peculiar appropriateness and efficacy in cases of extreme exhaustion of the system, where deglutition is difficult or impossible, or where the stomach rejects everything; where any other mechanical application to the uterus is dangerous or inconvenient, and especially where the introduction of the hand into the uterus would be likely to be attended by injury or even a fatal result. Indeed, it may be truly said that in cases of extreme exhaustion galvanism is the last resource left to us. The galvanic stimulus can be applied when every thing besides is out of the question. The uterine muscular fibre will respond to this stimulus when the nervous system is utterly prostrate, when the heart has ceased to beat, when the patient is moribund or even dead.

5th. Galvanism is less exhausting to the system than ergot, or most other means of exciting contraction. It acts directly upon the uterine muscular fibre, and scarcely taxes at all the general powers of the system.

6th. It does not necessarily preclude or supersede the use of other remedies tending to fulfil the same indication.*

*Devonshire-square*, October, 1853.

*An apparatus which combines to the greatest extent compactness, portability, and efficiency, is an especial desideratum to the obstetric practioner. I know of no instrument that possesses these advantages to the same extent as that of Mr. Hearder, of Plymouth. This instrument is well made, it does not occupy one-half the space of those usually sold, and while the maximum power is considerably greater, it admits, by the most simple means, of accurate and minute graduation. This last quality, independent of its obvious utility in regulating the power according to the effect desired, is one which the scientific practitioner will appreciate as affording the means of comparing his own observations with those of others. Further improvements in this machine are being effected, which will carry compactness to the furthest extent, and render it in every way admirably suited to the obstetric practitioner.

